# Alleviation of Adipose Tissue Inflammation and Obesity Suppression by a Probiotic Strain That Induces GLP-1 Secretion

**DOI:** 10.3390/microorganisms13061211

**Published:** 2025-05-26

**Authors:** A-Ram Kim, Seong-Gak Jeon, So-Jung Park, Heeji Hong, Byung Kwon Kim, Hyung-Ran Kim, Chun-Pyo Hong, Bo-Gie Yang

**Affiliations:** 1 Research Institute, GI Biome Inc., Seongnam 13201, Republic of Korea; arkim@gi-biome.com (A.-R.K.); sgjeon@gi-biome.com (S.-G.J.); sjpark@gi-biome.com (S.-J.P.); heeji.hong@gi-biome.com (H.H.); bkkim@gi-biome.com (B.K.K.); hrkim@gi-biome.com (H.-R.K.); 2 Research Institute, GI Cell Inc., Seongnam 13201, Republic of Korea; chong@gi-cell.com

**Keywords:** probiotics, *Lactiplantibacillus plantarum*, glucagon-like peptide-1, anti-inflammation, obesity

## Abstract

Glucagon-like peptide-1 (GLP-1) is a hormone secreted from enteroendocrine cells that can promote weight loss and blood glucose improvement. We screened probiotic strains that effectively stimulate GLP-1 secretion from human enteroendocrine cells and then investigated the efficacy of this strain in a high-fat diet (HFD)-induced mouse model of obesity. *Lactiplantibacillus plantarum* GB104 greatly induced GLP-1 secretion by increasing expression of the proglucagon gene (*GCG*), but not the proprotein convertase subtilisin/kexin type 1 gene (*PCSK1*) in the human enteroendocrine cell line NCI-H716. In an HFD-induced mouse model of obesity, GB104 inhibited weight gain and improved blood glucose levels by increasing blood GLP-1 levels. It also tended to attenuate the HFD-induced changes in blood levels of other hormones and suppressed fat accumulation in the liver and adipose tissues. In white adipose tissue, GB104 suppressed inflammation by reducing pro-inflammatory M1 macrophages and increasing anti-inflammatory M2 macrophages and regulatory T cells. Probiotic strains that promote GLP-1 secretion, such as GB104, may serve as a promising candidate for dietary intervention against obesity and metabolic diseases.

## 1. Introduction

Obesity involves a low-level chronic inflammatory state driven by the accumulation of pro-inflammatory M1 macrophages expressing CD11c in adipose tissue, and this inflammation can contribute to the development of various metabolic diseases and complications [1]. However, in the lean state, adipose tissue contains higher numbers of anti-inflammatory M2 macrophages expressing CD206 [2,3]. The progression to obesity not only switches the characteristics of adipose tissue macrophages from the anti-inflammatory M2 to pro-inflammatory M1 type but also features a reduction in regulatory T (Treg) cells [4]. Adipose tissue can induce systemic inflammation by increasing the secretion of inflammatory adipokines (e.g., resistin and PAI-1) and decreasing the secretion of anti-inflammatory adipokines like adiponectin [5,6]. The inflammation arising with obesity can be alleviated by diet intervention and weight loss [7].

The human body secretes various hormones to maintain metabolic homeostasis. Hormones such as glucagon-like peptide-1 (GLP-1), gastric inhibitory polypeptide (GIP), and ghrelin are secreted from gastrointestinal tissue to influence appetite regulation and insulin secretion [8]. Leptin secreted from adipose tissue also plays a role in appetite control [9]. Of these hormones, GLP-1 and leptin act to suppress appetite, while ghrelin acts to stimulate appetite. However, a high-fat diet (HFD) induces leptin and ghrelin resistance, which results in increased blood leptin levels and reduced blood ghrelin levels [10,11]. GIP promotes insulin and glucagon secretion and is involved in fat accumulation [12], with blood levels of this hormone increasing with an HFD [13]. On the other hand, insulin and glucagon, which are hormones secreted from the pancreas, act to lower and raise blood glucose levels, respectively [14]. HFD-induced obesity reduces insulin sensitivity, which increases its blood levels [15]. Metabolic homeostasis is normally maintained by these various hormones, thereby inhibiting the development of obesity and metabolic diseases.

Recently, GLP-1 receptor agonists (GLP-1RA) such as liraglutide, dulaglutide, and semaglutide have seen widespread use as treatments for obesity and metabolic diseases [16]. GLP-1 contributes to weight loss and blood glucose improvement by binding to GLP-1R, a G protein-coupled receptor (GPCR) [17]. GLP-1 is produced mainly by enteroendocrine L cells, pancreatic α cells, and the nucleus tractus solitary (NTS) of the brain following cleavage of proglucagon (*GCG*) by enzymes such as proprotein convertase subtilisin/kexin type 1 and type 2 (*PCSK1* and *PCSK2*) [18]. *PCSK1* acts primarily in the intestine and brain, whereas *PCSK2* acts in the pancreas. Additionally, while the appetite suppressing effect of GLP-1 occurs in the central nervous system (CNS), its insulin-secreting action occurs in pancreatic β cells [16,19]. Endogenous GLP-1 has a short plasma half-life due to its degradation by dipeptidyl peptidase-4 (DPP4) [18]. For this reason, GLP-1RAs are fundamentally designed to be difficult for DPP4 to act on, resulting in efficacy that is much stronger than endogenous GLP-1 [20]. However, if GLP-1RA administration is discontinued without a simultaneous improvement in lifestyle and dietary habits, weight regain may occur, and metabolic symptoms can become worse than before administration [21,22]. Furthermore, there are limits to continuous administration due to economic burden and injection aversion.

The gut microbiota is in close contact with intestinal epithelial cells and can stimulate enteroendocrine cells to secrete GLP-1 [23]. We sought to identify lactic acid bacterial strains achieving this effect. Promising strains that promote GLP-1 secretion are expected to provide useful options for the prevention and treatment of obesity and metabolic diseases, as they may be taken orally throughout life with lower economic burden.

## 2. Materials and Methods

### 2.1. Preparation of Cell-Free Culture Supernatant (CFS) of Lactic Acid Bacteria

Lactic acid bacteria were inoculated into 10 mL tubes containing 7 mL of MRS medium and cultured in an anaerobic Whitley A45 workstation (Don Whitley Scientific, Bingley, UK) using mixed gas (N_2_:H_2_:CO_2_ = 90:5:5) for 16 h. To prepare the CFS, centrifugation (10,000× *g* for 10 min) and 0.2 μm filtration were used. The sources of lactic acid bacteria used in the experiment are as follows. *Limosilactobacillus fermentum* GB102 (=MG4261) and GB103 (=MG4227) and *Lactiplantibacillus plantarum* GB104 (=MG4270) were isolated from the vagina of a healthy woman [24], and *Bifidobacterium longum* GB301 (=KACC91532) was isolated from the feces of a healthy infant [25]. *Lactiplantibacillus plantarum* WCFS1 (same as ATCC BAA-793 strain) was purchased from the ATCC (American Type Culture Collection) and *Lacticaseibacillus rhamnosus* GG (same as KCTC 5033 strain; LGG) was purchased from the KCTC (Korean Collection for Type Cultures).

### 2.2. In Vitro Assay for GLP-1 Secretion

To examine the ability of lactic acid bacteria to stimulate GLP-1 secretion, the human enteroendocrine cell line NCI-H716 was used, which was cultured in RPMI1640 medium (LM011-01; WELGENE, Gyeongsan, Republic of Korea) supplemented with 10% fetal bovine serum (17974671; GIBCO, Waltham, MA, USA) and 1% penicillin-streptomycin (LS202-02; WELGENE, Gyeongsan, Republic of Korea). For the experiment, NCI-H716 cells were seeded in 96-well plates at 3 × 10^5^ cells/well, and the following day, the culture medium was changed to HBSS containing 0.2% bovine serum albumin (BSA; A0100; GenDEPOT, Baker, TX, USA). After 2 h of incubation, NCI-H716 cells were stimulated with bacterial CFS for 2 h. Culture supernatant from the NCI-H716 cells was harvested and stored at −80 °C until GLP-1 concentration measurement. GLP-1 measurements were performed using the RayBio^®^ GLP-1 Enzyme Immunoassay Kit (EIA-GLP; RayBiotech, Peachtree Corners, GA, USA) according to the manufacturer’s instructions.

### 2.3. RNA Extraction and Quantitative RT-PCR (qRT-PCR) Analysis

Total RNA was extracted from NCI-H716 cells stimulated with each bacterial CFS for 2 h using the Easy-spin Total RNA Extraction Kit (17221; iNtRON Biotechnology, Seongnam, Republic of Korea). cDNA was synthesized from the extracted RNA using the SuPrimeScript cDNA Synthesis Kit (SRK-1000; Genetbio, Daejeon, Republic of Korea). RT-qPCR was carried out on a QuantStudio^™^ 3 Real-Time PCR System (Applied Biosystems, Foster City, CA, USA) using the AccuPower^®^ 2X GreenStar^™^ qPCR Mix (K-6251; Bioneer, Daejeon, Republic of Korea). All experiments were performed according to the manufacturer’s instructions. The thermal cycling conditions were as follows: initial hold at 95 °C for 5 min, followed by 40 cycles of 95 °C for 15 s and 60 °C for 10 s. The expression levels of individual genes were normalized to the expression of the housekeeping gene GAPDH. The primer sequences used for each gene are shown in Table 1.

### 2.4. Acid Resistance Test

Based on a previous report [26], acid resistance tests of GB104 were performed at various pH levels and bile acid contents. LGG was used as a control strain. For the experiment, MRS medium was adjusted to a specific pH with HCl and NaOH or for a specific bile acid content by adding oxgall (BD Difoco, Franklin Lakes, NJ, USA). GB104 were inoculated into the MRS broth and cultured anaerobically at 37 °C for 4 h, and the number of viable cells was measured at 2 h intervals during culture. Viable cell counts were measured using a spread plate method and expressed as colony forming units (CFUs). In addition, to examine acid resistance in artificial gastrointestinal fluids mimicking the gastrointestinal environment, GB104 was incubated in pH 3.0 artificial gastric juice at 37 °C for 2 h and then incubated in pH 8.0 artificial intestinal juice at 37 °C for 4 h. The artificial gastric juice (Biochemazone, Leduc, AB, Canada) was adjusted to a final pH of 3.0 with NaOH, and the artificial intestinal juice (Biochemazone, Leduc, AB, Canada) containing 0.3% oxgall (BD Difoco, Franklin Lakes, NJ, USA) and 0.1% pancreatin (Sigma-Aldrich, St. Louis, MO, USA) was adjusted to a final pH of 8 with NaOH. When changing from artificial gastric juice to artificial intestinal juice, GB104 was harvested by centrifugation (8000 rpm for 5 min) and washed with PBS. Viable cell counts were measured at 2 h intervals during culture.

### 2.5. HFD-Induced Mouse Model of Obesity

The animal experiments were performed in the animal facilities of Seoul National University Institute of Systems Immunology (Hongcheon, Republic of Korea) and GI Biome Inc. (Seongnam, Republic of Korea), with approval from the Institutional Animal Care and Use Committee (IACUC; approval code: SNU-190212 (12 February 2019), GIB-24-05-003 (3 June 2023)). Five-week-old male C57BL/6 mice were purchased from Orient Bio (Seongnam, Republic of Korea) and acclimatized for 1 week before the experiment. The animals were housed under specific-pathogen-free (SPF) conditions and maintained in a temperature-controlled environment with a 12 h dark/light cycle. For the experiment, mice were randomly assigned to groups with comparable average body weights. Mice in the high-fat diet (HFD) group were fed a 60 kcal% fat diet (D12492; research diet, New Brunswick, NJ, USA) or 45 kcal% fat diet (D12451; research diet), while those in the normal chow diet (NCD) group received a normal chow diet (Teklad Global 18% Protein Rodent Diet 2018S) (Inotiv, Lafayette, IN, USA). GB104 was administered orally once daily, starting concurrently with the initiation of the HFD. The strain was cultured and lyophilized by Mediogen (Jecheon, Republic of Korea), then resuspended in phosphate-buffered saline (PBS) and administered at 5 × 10^9^ CFU/200 µL per mouse using a flexible disposable feeding needle (JD-S-126-5202; JEUNGDO Bio & Plant, Seoul, Republic of Korea). Weight gain was measured weekly. In another experiment, HFD mice were fed a 45 kcal% fat diet (D12451; research diet, New Brunswick, NJ, USA), and GB104 was orally administered at 1 × 10^9^ CFU/200 µL per mouse for 8 weeks, starting from 5 weeks after HFD initiation.

### 2.6. Glucose Tolerance Test (GTT)

GTTs were performed after the mice fasted for 16 h. After measuring fasting blood glucose levels, the mice were intraperitoneally administered a glucose solution at 1 g/Kg body weight, and blood glucose levels were measured from the tail vein using an Auto-chek Plus glucose meter (GM01RAA; i-SANS, Seoul, Republic of Korea) at 15, 30, 60, and 120 min. The area under the curve (AUC) of the graph showing blood glucose levels over time was calculated using GraphPad Prism version 9.5.1 (GraphPad Software, La Jolla, CA, USA).

### 2.7. Insulin Tolerance Test (ITT)

ITTs were performed after the animals fasted for 4.5 h. After measuring blood glucose levels, the mice were intraperitoneally administered an insulin solution at 1 U/Kg body weight, and blood glucose levels were measured from the tail vein using an Auto-chek Plus glucose meter (GM01RAA; i-SANS, Seoul, Republic of Korea) at 15, 30, 60, and 120 min. The area under the curve (AUC) of the graph showing blood glucose levels over time was calculated using GraphPad Prism version 9.5.1 (GraphPad Software, La Jolla, CA, USA).

### 2.8. Measurement of Metabolic Hormone Levels in Serum

The concentrations of metabolic hormones in mouse serum were measured using the Bio-plex Pro Mouse Diabetes 8-plex assay kit using the Bio-Plex 200 system (Bio-Rad, Hercules, CA, USA). All experiments were performed according to the manufacturer’s instructions.

### 2.9. Histological Analysis

To visualize fat accumulation in the mouse liver and adipose tissues, tissue samples were fixed in 10% neutral buffered formalin (NBF) to prepare paraffin sections, followed by hematoxylin and eosin (H&E) staining. All experiments for histological analysis were performed at Seoul National University Institute of Systems Immunology (Hongcheon, Republic of Korea).

### 2.10. Isolation of Immune Cells in Adipose Tissue

Isolation of immune cells from epididymal adipose tissue was performed as previously reported [27]. Briefly, epididymal adipose tissue samples were minced and then digested in enzyme media (RPMI1640 media containing 400 U/mL collagenase D, 10 µg/mL DNase I, 1 mM sodium pyruvate, and 1 mM NEAA) for 45 min at 37 °C. The enzyme reaction was stopped by adding EDTA to a final concentration of 10 mM. After 40 um filtration, immune cells were enriched by 40/75% Percoll (Cytiva, Marlborough, MA, USA) gradient centrifugation.

### 2.11. Immune Cell Analysis by Flow Cytometry

The antibodies used to identify immune cells in this study were fluorescently labeled antibodies recognizing the following targets: MHCII (M5/114.15.2), F4/80 (BM8), CD11b (M1/70), CD11c (HL3), CD206 (C068C2), TCRβ (H57-597), CD4 (RM4-5), and Foxp3 (FJK-16s). In all experiments, Fc receptors were blocked with anti-CD16/CD32 antibodies (TruStain FcXTM, BioLegend, San Diego, CA, USA) before staining of the immune cells. Macrophages were stained using antibodies recognizing MHCII, F4/80, CD11b, CD11c, and CD206. For Treg cell staining, we first stained TCRβ and CD4 on the cell surface and then stained for Foxp3 in the nucleus using Foxp3 staining buffer solution (eBioscience, San Diego, CA, USA). The stained cells were analyzed using LSRFortessa (BD Biosciences, Franklin Lakes, NJ, USA) and FlowJo software (v10.10.0, Tree Star, San Carlos, CA, USA).

### 2.12. Statistical Analysis

Statistical analyses of all data were performed using GraphPad Prism version 9.5.1 (GraphPad Software, La Jolla, CA, USA). Data were statistically analyzed using the two-tailed unpaired Student *t*-test. Statistical significance based on *p*-values is indicated in each graph as follows: * *p* < 0.05, ** *p* < 0.01, *** *p* < 0.001, **** *p* < 0.0001.

## 3. Results

### 3.1. GB104 Promotes GLP-1 Secretion from Enteroendocrine Cells and Exhibits Similar Acid Resistance to LGG

**Figure 1:**
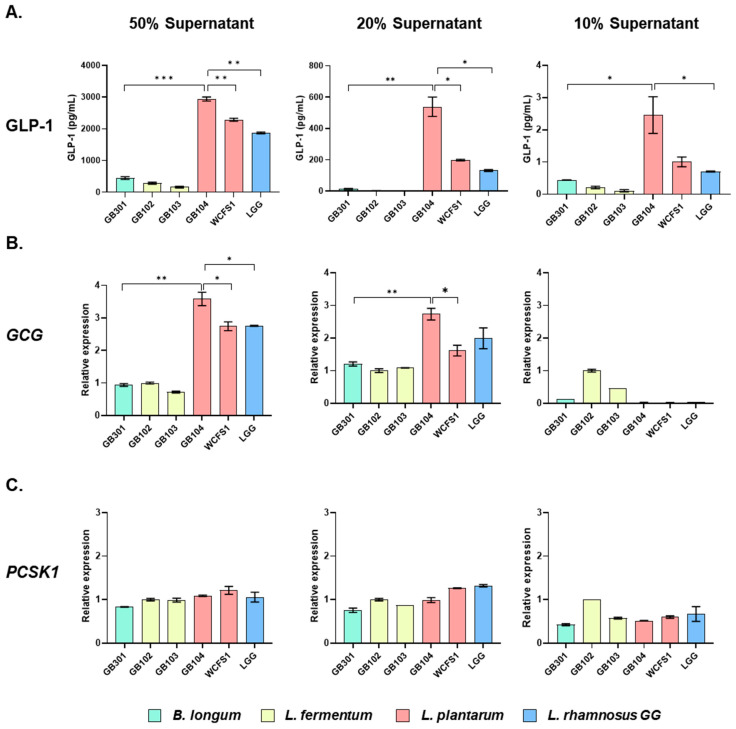
Effect of CFS of lactic acid bacteria on GLP-1 secretion and related gene expression. After treating NCI-H716 cells with CFS of each lactic acid bacterial strain at concentrations of 50%, 20%, and 10%, secreted GLP-1 levels (**A**) and relative expression levels of *GCG* (**B**) and *PCSK1* (**C**) were examined. All data are presented as mean ± SD. * *p* < 0.05; ** *p* < 0.01; *** *p* < 0.001.

To select probiotic strains that effectively promote GLP-1 secretion, the human enteroendocrine cell line NCI-H716 was treated with the cell-free supernatant (CFS) of lactic acid bacteria at concentrations of 50%, 20%, and 10% for 2 h. Then, GLP-1 secreted by the NCI-H716 cells was measured by ELISA. *L. plantarum* GB104 showed the most striking ability to stimulate GLP-1 secretion, followed by *L. plantarum* WCFS1 and *L. rhamnosus* GG (LGG) (Figure 1A). In particular, the differences between strains were most evident when 20% CFS was used. Since GLP-1 is produced in enteroendocrine cells by cleavage of *GCG* by *PCSK1* [18], we investigated whether the CFS of each strain affected the expression of these genes. The CFS of GB104 significantly increased *GCG* expression at 50% and 20%, but not at 10% dilution (Figure 1B). Unlike *GCG* expression, *PCSK1* expression was not affected by the CFS of GB104 (Figure 1C). These results demonstrate that GB104 promotes GLP-1 secretion by increasing *GCG* expression, but not *PCSK1* expression.

**Figure 2:**
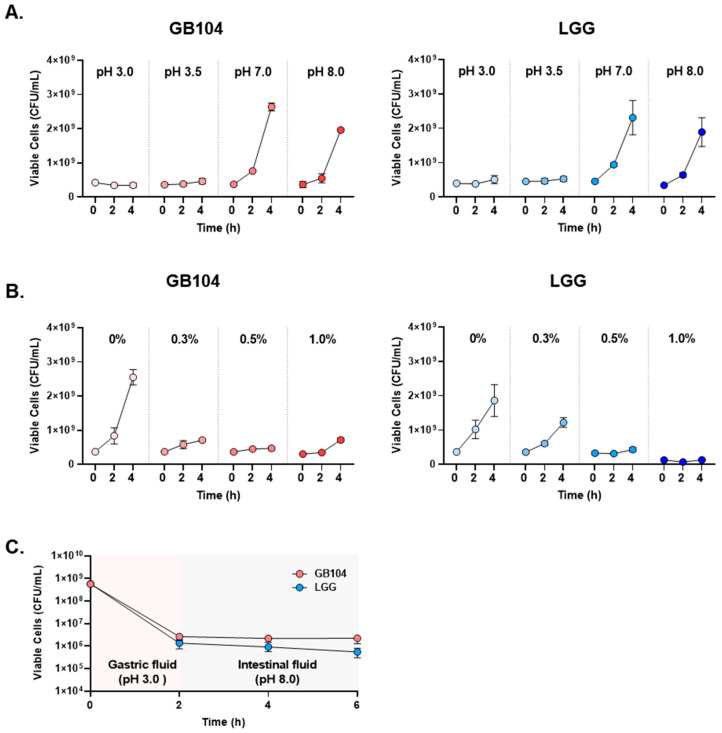
Comparison of acid resistance between *L. plantarum* GB104 and LGG. (**A**,**B**) To compare the acid resistance between *L. plantarum* GB104 (red) and LGG (blue), experiments were performed under various pH and bile acid content conditions. (**C**) In addition, to compare acid resistance in artificial gastrointestinal fluids mimicking the gastrointestinal environment, each strain was sequentially cultured in artificial gastric fluid (pH 3.0) and artificial intestinal fluid (pH 8.0). All data are presented as mean ± SD.

In addition, since GB104 is administered orally, its acid resistance was compared with LGG, which is widely used industrially. GB104 showed similar acid resistance to LGG at all pH levels and with varying bile acid content (Figure 2A,B). Moreover, in artificial gastrointestinal fluids mimicking the gastrointestinal environment, GB104 appeared slightly superior to LGG (Figure 2C). Therefore, the possibility of GB104 passing through the stomach and reaching the intestine alive is likely to be similar to that of LGG.

### 3.2. GB104 Increases Blood GLP-1 Levels and Inhibits Weight Gain in an HFD-Induced Mouse Model of Obesity

**Figure 3:**
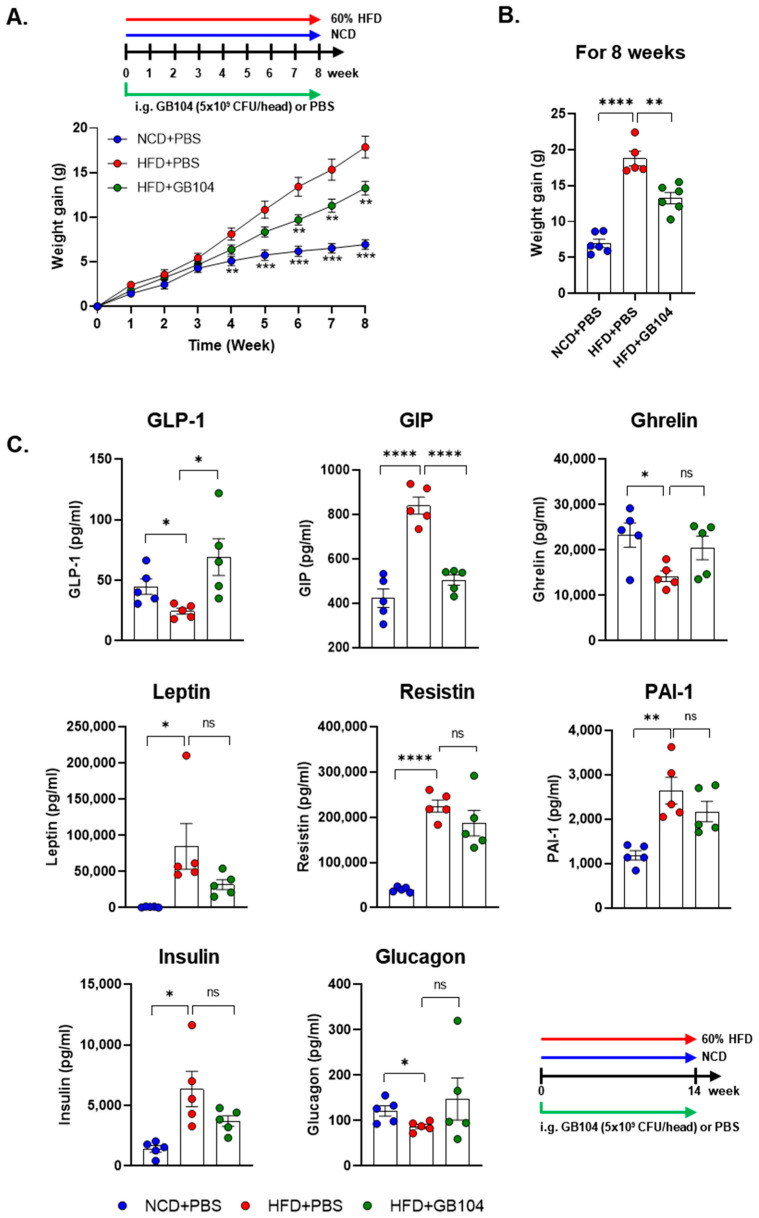
Effects of *L. plantarum* GB104 on weight gain and blood hormones in an HFD-induced obese mouse model. To determine whether *L. plantarum* GB104 suppresses weight gain, the body weights of mice were measured weekly (**A**), with the changes over 8 weeks presented as a bar graph (**B**). In addition, changes in blood hormone levels elicited by *L. plantarum* GB104 were examined (**C**). Mice used in this experiment were fed a 60% HFD for 14 weeks. Statistical analyses of the graph in (**A**) were performed relative to the HFD + PBS control group. NCD, normal chow diet; HFD, high-fat diet. All data are presented as mean ± SEM (*n* = 5–6 per sample). * *p* < 0.05; ** *p* < 0.01; *** *p* < 0.001; **** *p* < 0.0001; ns, not significant.

GB104 significantly suppressed weight gain in an HFD-induced mouse model of obesity (Figure 3A,B). We also investigated whether blood levels of various metabolic hormones and adipokines were affected by GB104 using the Bio-plex Pro Mouse Diabetes 8-plex assay kit. The in vivo experimental result showed that the level of GLP-1 in blood was significantly increased by GB104 (Figure 3C). Metabolic hormones such as GIP, leptin, and insulin are known to increase by HFD [13,15], which was also observed in our experiment (Figure 3C). Among these hormones, blood levels of GIP were most clearly reduced by GB104 administration (Figure 3B). On the other hand, the blood levels of leptin and insulin showed a tendency to decrease following GB104 administration, but this was not statistically significant (Figure 3C). In addition, reductions in ghrelin and glucagon levels induced by HFD showed a tendency to increase after GB104 treatment, but this also failed to achieve statistical significance (Figure 3C). Pro-inflammatory adipokines such as resistin and PAI-1 were also not significantly reduced by *L. plantarum* GB104 (Figure 3C).

### 3.3. GB104 Improves Blood Glucose Levels and Suppresses Fat Accumulation

**Figure 4:**
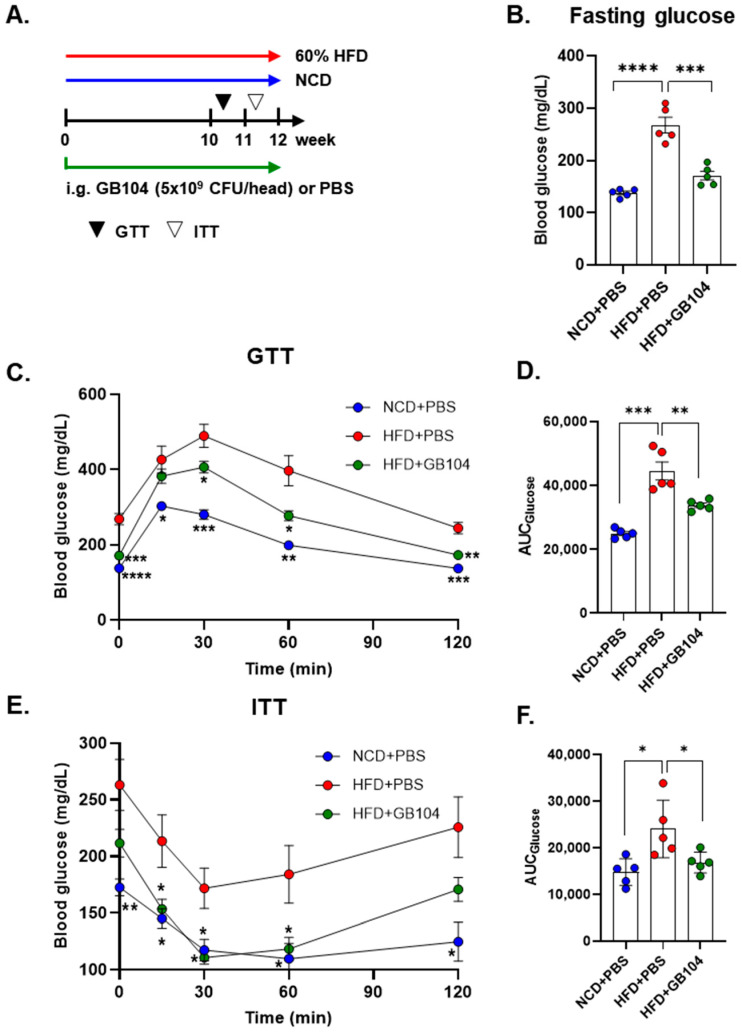
Effect of *L. plantarum* GB104 on blood glucose improvement in an HFD-induced obese mouse model. To investigate the effect of *L. plantarum* GB104 on blood glucose levels, GTT (**B**–**D**) and ITT (**E**,**F**) were performed according to the schedule outlined in the experimental design diagram (**A**). The experiments were performed after fasting, and glucose and insulin were administered intraperitoneally. Fasting blood glucose levels were measured before the GTT (**B**). The area under the curves (AUCs) of the GTT and ITT graphs were calculated to compare between groups. Statistical analyses of the graphs in (**C**,**E**) were performed relative to the HFD + PBS control group. NCD, normal chow diet; HFD, high-fat diet. All data are presented as mean ± SEM (*n* = 5–6 per sample). * *p* < 0.05; ** *p* < 0.01; *** *p* < 0.001; **** *p* < 0.0001.

**Figure 5:**
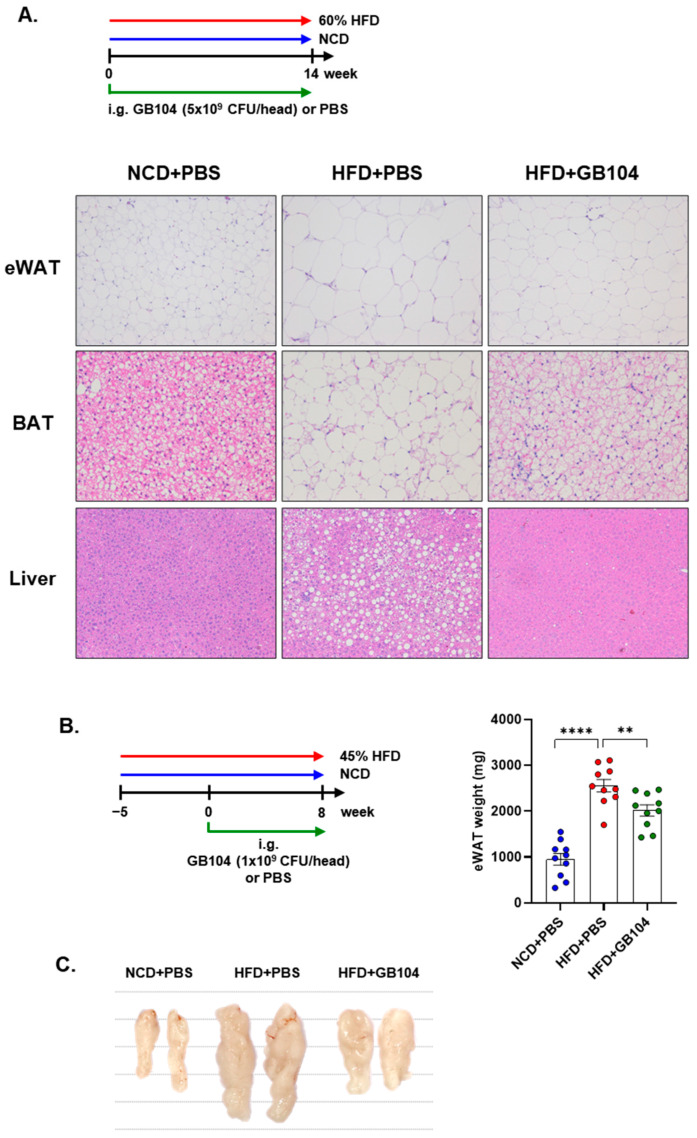
Effects of *L. plantarum* GB104 on fat accumulation in an HFD-induced obese mouse model. To determine whether *L. plantarum* GB104 inhibits fat accumulation, paraffin sections of liver and adipose tissues from mice fed a 60% HFD for 14 weeks were stained with H&E. Representative images are presented (**A**). Liver and epididymal white adipose tissue (eWAT) were observed at 100× magnification, and brown adipose tissue (BAT) was observed at 200× magnification. In addition, *L. plantarum* GB104 was orally administered daily to mice fed a 45% HFD for 8 weeks, and then the weights of eWAT samples were measured (**B**). Representative eWAT samples are presented in the photographic image (**C**). NCD, normal chow diet; HFD, high-fat diet. All data are presented as mean ± SEM (*n* = 10 per sample). ** *p* < 0.01; **** *p* < 0.0001.

GB104 was shown to significantly reduce fasting blood glucose levels (Figure 4B) as well as blood glucose levels following a glucose tolerance test (GTT) (Figure 4C,D) in an HFD-induced mouse model of obesity. An insulin tolerance test (ITT) showed that the impairment of insulin sensitivity in this model was recovered by GB104 (Figure 4E,F). In addition, histochemical staining images showed that the size of fat droplets in liver and adipose tissue samples (epididymal white adipose tissue and brown adipose tissue) was reduced by GB104 (Figure 5A). Supporting these findings, GB104 also significantly reduced the mass of epididymal adipose tissue (Figure 5B,C).

### 3.4. GB104 Alleviates Adipose Tissue Inflammation Induced by HFD

**Figure 6:**
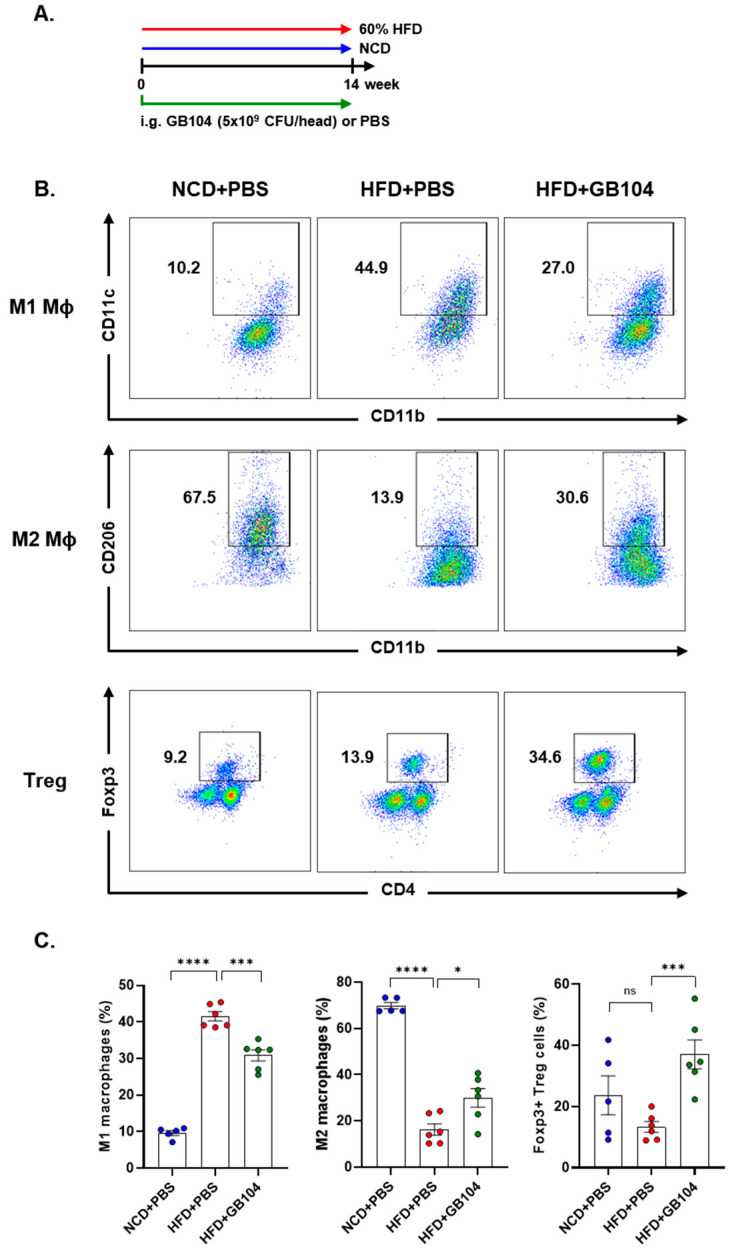
Effect of *L. plantarum* GB104 on immune cells in adipose tissue. Animal experiments were conducted according to the experimental design diagram (**A**). Subsequently, immune cells were then isolated from epididymal adipose tissues, and M1 and M2 macrophages, as well as Treg cells, were analyzed using flow cytometry. Representative results are presented as scatter plots (**B**), and overall results are presented as individual bar graphs (**C**). After gating the MHCII^+^F4/80^+^ cell population, M1 macrophages were identified as the CD11c^+^CD11b^+^ cell population, while M2 macrophages were identified as the CD11b^+^CD206^+^ cell population. Treg cells were identified as the CD4^+^Foxp3^+^ cell population after gating the TCRβ^+^ cell population. NCD, normal chow diet; HFD, high-fat diet. * *p* < 0.05; *** *p* < 0.001; **** *p* < 0.0001; ns, not significant.

Obesity is defined as a low-level chronic inflammatory state, and suppression of inflammation is important for the prevention and treatment of metabolic diseases [1]. We analyzed immune cells in adipose tissue to determine whether GB104 alleviates the inflammation induced by HFD (Figure 6A). Macrophages were identified as the MCHII^+^F4/80^+^CD11b^+^ cell population. After gating the MCHII^+^F4/80^+^ cell population, M1 macrophages were identified as the CD11c^+^CD11b^+^ cell population, and M2 macrophages were identified as the CD206^+^CD11b^+^ population (Figure 6B). Treg cells were identified as the Foxp3^+^CD4^+^ population after gating the TCRβ^+^ T cells (Figure 6B). As expected, HFD significantly increased M1 macrophages and decreased M2 macrophages, thereby inducing adipose tissue inflammation, which was significantly inhibited by GB104 (Figure 6B,C). On the other hand, Treg cells were not significantly affected by HFD but were greatly increased by GB104 (Figure 6B,C). These results demonstrate that GB104 alleviates HFD-induced inflammation in adipose tissue by reducing pro-inflammatory M1 macrophages and increasing anti-inflammatory M2 macrophages and Treg cells.

## 4. Discussion

In this study, we found that *L. plantarum* GB104 effectively promotes GLP-1 secretion in vitro using the enteroendocrine cell line NCI-H716. Cell-free culture supernatant (CFS) from this strain effectively enhanced GLP-1 secretion by upregulating the expression of the *GCG* gene (Figure 1A,B). However, it did not significantly affect the expression of *PCSK1*, an enzyme that cleaves *GCG* to produce GLP-1 (Figure 1C). These results suggest that metabolites produced by GB104 primarily enhance GLP-1 secretion through the upregulation of *GCG* gene expression in enteroendocrine cells. Future studies are required to identify the metabolites involved in this process. It should also be investigated whether these metabolites affect the activity of DPP4, a GLP-1-degrading enzyme, as well as the expression of GLP-1 receptor.

GB104 administration increased blood GLP-1 levels and contributed to weight suppression and blood glucose improvement in an HFD-induced obese mouse model (Figure 3A,B and Figure 4). In addition, GB104 significantly reduced circulating levels of the incretin hormone GIP (Figure 3C). GIP promotes insulin secretion like GLP-1 but is also involved in fat accumulation [12]. Given this fact, it appears likely that the GIP reductions induced by GB104 contribute to a reduction in adipocyte size and tissue mass. GB104 also significantly increased insulin sensitivity (Figure 4E,F) but did not significantly reduce blood insulin levels (Figure 3C). Since GLP-1 is characterized as an anti-inflammatory factor [28], we investigated whether GB104 inhibits HFD-induced inflammation in adipose tissue and found a significant reduction in pro-inflammatory M1 macrophages, with a concurrent increase in anti-inflammatory M2 macrophages and Treg cells (Figure 6). However, the blood levels of inflammatory adipokines such as resistin and PAI-1 were not significantly reduced (Figure 3C). Of note, GB104 shows comparable acid resistance to LGG, which is widely used industrially (Figure 2). Therefore, it is expected that *L. plantarum* GB104 can contribute to the prevention and treatment of obesity and metabolic diseases by promoting GLP-1 secretion from enteroendocrine cells.

Recently, GLP-1RAs such as liraglutide, dulaglutide, and semaglutide have seen widespread use as treatments for obesity and diabetes [16], but issues for patients remain, including economic burden and injection aversion. To address these issues, small molecule GLP-1RAs that can be administered orally, such as orforglipron, are being developed [16]. However, small molecules can come with a high incidence of side effects including nausea and vomiting [29,30,31], and new patient-friendly approaches are still needed. From this perspective, probiotics such as GB104 may be a good solution. Orally administered GB104 stimulates enteroendocrine cells via its metabolites, leading to enhanced endogenous GLP-1 secretion. However, the secreted GLP-1 is rapidly degraded by the DPP4 enzyme and has a short half-life [18], which may limit its efficacy compared to GLP-1RAs that are more resistant to DPP4. Therefore, to achieve therapeutic effects, probiotics that promote GLP-1 secretion may require daily oral administration over extended periods. Despite this, such probiotics are generally safe and cost-effective for long-term use and may also contribute to healthier diet and lifestyle habits. Because the effectiveness of these probiotic strains in humans is not yet established, future clinical trials are needed. Moreover, to observe meaningful effects in populations with diverse diets and lifestyles, larger-scale studies will be necessary comparing animal studies involving inbred mice on controlled diets. In contrast, although GLP-1RAs are highly effective, discontinuation without concurrent lifestyle improvements may result in rebound weight gain and worsening blood glucose levels. Considering these factors, probiotic strains that promote GLP-1 secretion may serve as valuable complementary or alternative strategies for the prevention and treatment of obesity and metabolic diseases alongside GLP-1RAs.

## 5. Conclusions

*L. plantarum* strain GB104, which promotes GLP-1 secretion from enteroendocrine cells, increases blood GLP-1 levels, improves blood glucose levels, and suppresses weight gain in HFD-induced obese mice. GB104 also reduces the number of pro-inflammatory M1 macrophages and increases the number of anti-inflammatory M2 macrophages and Treg cells in adipose tissue, thereby alleviating HFD-induced inflammation. Due to their ease of administration and lower risk of side effects, promising probiotic strains like GB104 may soon find applications in the prevention and treatment of obesity and metabolic diseases.

## 6. Patents

*L. plantarum* GB104 is patent pending in the Republic of Korea (10-2023-0090950), and a PCT application exists (PCT/KR2020/019347).

## Figures and Tables

**Table 1:** Primer sequences used in qRT-PCR.

Gene	Forward Sequence (5′ to 3′)	Reverse Sequence (3′ to 5′)
*hG* *APDH*	GGAGCGAGATCCCTCCAAAAT	GGCTGTTGTCATACTTCTCATGG
*hGCG*	ACCAGAAGACAGCAGAAATG	GAATGTGCCCTGTGAATG
*hPCSK1*	CAGAAGGCTTTTGAATATGGTGT	GGAGGCACTGCTGATGGAGAT

## Data Availability

The original contributions presented in this study are included in the article; further inquiries can be directed toward the corresponding author.

## Abbreviations

The following abbreviations are used in this manuscript:

GLP-1	glucagon-like peptide-1
HFD	high-fat diet
GLP-1RA	GLP-1 receptor agonist
GPCR	G protein coupled receptor
*GCG*	proglucagon gene
PCSK	proprotein convertase subtilisin/kexin
GIP	gastric inhibitory polypeptide
Treg	regulatory T cells
CNS	central nervous system
NTS	nucleus tractus solitary
CSF	cell-free culture supernatant
GAPDH	glyceraldehyde-3-phosphate dehydrogenase
ATCC	American Type Culture Collection
KCTC	Korean Collection for Type Cultures
GTT	glucose tolerance test
ITT	insulin tolerance test
NBF	neutral buffered formalin
H&E	hematoxylin and eosin
NEAA	non-essential amino acids
EDTA	ethylenediaminetetraacetic acid
MHC	major histocompatibility complex
